# Smoking Intensity and Associated Factors among Male Smokers in Ethiopia: Further Analysis of 2016 Ethiopian Demographic and Health Survey

**DOI:** 10.1155/2020/4141370

**Published:** 2020-07-23

**Authors:** Simegnew Handebo, Setognal Birara, Ayenew Kassie, Adane Nigusie, Wallelign Aleminew

**Affiliations:** ^1^Department of Health Education and Behavioral Sciences, Institute of Public Health, College of Medicine and Health Sciences, University of Gondar, Gondar, Ethiopia; ^2^Department of Public Health, College of Medicine and Health Sciences, Samara University, Samara, Ethiopia

## Abstract

**Background:**

Smoking invariably has health, social, economic, and environmental consequences in Ethiopia. Reducing and quitting cigarette smoking improves individual health and increases available household funds for food, education, and better economic productivity. Therefore, this study is aimed at assessing cigarette smoking intensity and associated factors among male smokers in Ethiopia.

**Methods:**

The data were extracted from the 2016 national cross-sectional Ethiopian Demographic and Health Survey. Our study used data from the standardized and adapted men's questionnaire. The study included a total of 391 (weighted) smokers who at least smoked one manufactured cigarette per day. The data were collected using a two-stage cluster design which includes selection of enumeration areas and then selection of households. The number of manufactured cigarettes smokers smoked per day was used to measure smoking intensity. Descriptive statistics were used to summarize the study findings. Bivariable and multivariable truncated negative binomial Poisson regression models were employed to determine smoking intensity.

**Results:**

The finding showed that on average men smoked weighted nine cigarettes per day. One in every five of the smokers (21.2%) smoked 10 cigarettes per day. Smokers living in rural areas (IRR = 0.43, 95% CI: 0.244, 0.756), currently married (IRR = 0.64, 95% CI: 0.46, 0.91), formerly married (IRR = 0.54, 95% CI: 0.30, 0.96), richer men (IRR = 0.63, 95% CI: 0.43, 0.90), and richest men (IRR = 0.49, 95% CI: 0.28, 0.87) were associated with lower smoking intensity. Smokers in the Somali (IRR = 2.80, 95% CI: 1.29, 6.11), Harari (IRR = 3.46, 95% CI: 1.14, 10.51), and Dire Dawa (IRR = 3.09, 95% CI: 1.23, 7.80) regions; older age (IRR = 1.77, 95% CI: 1.31, 2.40); affiliated with Protestant religion (IRR = 1.81, 95% CI: 1.12, 2.92); poorer men (IRR = 1.64, 95% CI: 1.19, 2.27); watched television (IRR = 1.18, 95% CI: 1.04, 1.35); drunk alcohol (IRR = 1.37, 95% CI: 1.03, 1.82); and completed primary (IRR = 1.15, 95% CI: 1.01, 0.317) and higher education (IRR = 2.96, 95% CI: 1.88, 4.67) were positively associated with smoking intensity.

**Conclusion:**

Male smokers in Ethiopia smoked intensively with an average of nine manufactured cigarettes per day. Tobacco control interventions should target the following: Eastern Ethiopia regions, older aged, affiliated with Protestant religion, poorer men, watched television, drunk alcohol, and primary and higher educational level.

## 1. Introduction

Tobacco harms the health, the treasury, and the spirit of Ethiopia. Even though fewer men smoke in Ethiopia, people continue to die and become sick due to tobacco use. The costs associated with tobacco use are high in the society. There are more than 1.8 million men who smoke cigarettes each day. According to the World Health Organization (WHO) (2015), 8.9% of Ethiopian men aged 15 years and older smoke tobacco products. Every year, more than 16,800 Ethiopians are killed by tobacco-related diseases [1, 2].

Ethiopia has made progress on tobacco control in recent years [2]. The country became a part of the WHO Framework Convention on Tobacco Control on June 23, 2014, to address the challenges of tobacco control. The country passed an antitobacco bill in 2015 based on the Tobacco Control Directive (no. 28/2015) that includes measures governing tobacco smoke-free places and consumption, advertising, packaging, and labeling as well as taxation and prices [3].

Studies done in Ethiopia revealed that the majority of smokers were males. Studies in the country revealed that females smoked significantly lower than males [4–6]. For instance, in Addis Ababa, males smoke fourfold compared to females [7]. Similarly, a study in Harar town documented that 4% of females and 20.6% of males smoked cigarettes [8]. Likewise, at Mekelle University, 37.5% of males and 21.6% of females smoke cigarettes [9]. In general, females are restricted from smoking because of the low acceptance in the norms owing to familial responsibilities of caring for and feeding families. Female smoking might also be a reason for stigma and discrimination [4, 5, 8]. So, empirical analysis in this study was done solely on the male sample.

Studies on cigarette smoking in Ethiopia focused on its prevalence and predictors in specific groups, like university and high school students or specific areas [7–10]. Some studies utilized the EDHS data to determine factors associated with smoking. Two studies were done based on the 2011 EDHS data and focused on the prevalence and determinants of tobacco use and the effects of geographic variations [4, 11]. The other was based on the 2011 and 2016 EDHS data and assessed the geographic variations, the impact of khat (Catha edulis) chewing and other individual, and the household- and community-level determinants of smoking in Ethiopia [6].

Despite the presence of studies on tobacco use and associated factors, there is no study that assesses the smoking intensity among smokers. Evidence on smoking intensity is important for planning and implementing behavior change interventions in the country. Therefore, the current study focused on smoking intensity and associated factors among male smokers in Ethiopia.

## 2. Methods

### 2.1. Data and Participants

The study used the 2016 EDHS, the fourth countrywide population and health survey conducted by the Central Statistical Agency (CSA). It is a large cross-sectional household survey that uses a stratified two-stage cluster sampling method to collect nationally representative information. The survey was conducted from January 18, 2016, to June 27, 2016. More detailed information regarding the survey methods is presented in the EDHS report [12].

Our study used data from the standardized and adapted men's questionnaire. The male dataset of 2016 EDHS was accessed from the Measure DHS website (http://www.measuredhs.com) in STATA format. We extracted important variables based on the literature, the DHS recode-7 manual, and a questionnaire at the end of the report. The data were collected using a two-stage cluster design that includes selection of enumeration areas as a first stage and selection of households as a second stage. To account for the complex sampling design in the DHS, standard weight from the men's file was used to adjust for unequal probability of selection. Of the eligible men, 12,688 were successfully interviewed, with a response rate of 85.8% [12]. Of the total men included in the survey, 391 (weighted) who smoked at least one cigarette per day were included in the analysis.

### 2.2. Variables

Respondents who smoked a cigarette daily were asked the number of cigarettes they were smoking each day at the moment. Since a significant number of the daily smokers used manufactured cigarettes, this study adopted the number of manufactured cigarettes the participants used per day as an outcome variable to assess the smoking intensity among male smokers in Ethiopia. Based on the literature [4–11], age, marital status, residence, educational level, religion, occupational status, administrative region, wealth index, frequency of media use, current khat chewing behavior, and alcohol use were the selected independent variables.

### 2.3. Model Selection and Statistical Analysis

The classical Poisson regression model, commonly used to model count data, assumes the mean to be equal to the variance. The model fitness was assessed using the information criterion-based measures Akaike Information Criterion (AIC) and Bayesian Information Criterion (BIC). Smaller AIC and BIC values indicated a better model fit [13, 14]. The result from Table 1 reveals that of the five models suggested, the truncated negative binomial regression model is best fitted to assess factors associated with smoking intensity in Ethiopia (see Table 1). The statistical analysis was done using STATA version 14. The probability value of type 1 error less than 0.05 was considered statistically significant.

## 3. Results

### 3.1. Descriptive Statistics

Out of the total men interviewed in EDHS, 391 (weighted) of the observations were included in this study, as these were the observations with complete information for all variables of interest. Of the study participants, 76.7% were living in rural areas. Majority (82.4%) of them were married, and 69% were affiliated with Muslim religion. About half (48%) of the participants were in the 35-39 years age category. For the majority (61.5%) of the participants, agriculture was the main livelihood. With regard to media exposure, 46.45%, 38.45%, and 23.82% of the male smokers listened to the radio, watched television, and read newspapers, respectively. More than one in every four (26.1%) of the smokers were in the richest wealth category. Besides, 85.2% and 30.6% of the male smokers chew khat (Catha edulis) and drank alcohol, respectively (see Table 2).

### 3.2. Smoking Intensity

More than one in every five of the male smokers (21.2%) smoked 10 cigarettes per day. Smokers living in urban areas smoked 13.1 manufactured cigarettes daily. Smokers affiliated with Orthodox Christian religion smoked on average 11.4 cigarettes per day. Men who were employed in unskilled jobs smoked a mean of 14.3 manufactured cigarettes per day. Those who drank alcohol smoked 10.7 cigarettes per day. Overall, male smokers in Ethiopia smoked weighted nine manufactured cigarettes per day.

### 3.3. Factors Associated with Smoking Intensity

The finding revealed that smokers living in rural areas smoked 57% fewer cigarettes than those living in urban areas (IRR = 0.43; 95% CI: 0.244, 0.756). The older group (45–59 years) smoked 1.8 times more cigarettes than their younger counterparts (IRR = 1.77; 95% CI: 1.313, 2.399) keeping all other variables constant. Currently married (IRR = 0.64; 95% CI: 0.46, 0.91) and formerly married (IRR = 0.54; 95% CI: 0.30, 0.96) men smoke 36% and 46% fewer cigarettes than those who never married, respectively. Compared to men who are affiliated with Islam religious groups, those affiliated with Protestant religion were associated with having a higher smoking probability (IRR = 1.81; 95% CI: 1.12, 2.92). Smokers who completed primary (IRR = 1.15; 95% CI: 1.01, 0.317) and higher educational level (IRR = 2.96; 95% CI: 1.88, 4.67) smoked 1.39 and 2.96 times higher number of cigarettes than those who were illiterate, respectively.

Compared to smokers in the poorest wealth quartile, those who were in the poorer quartile (IRR = 1.64; 95% CI: 1.19, 2.27) smoked 1.64 times more cigarettes. Conversely, smokers in the richer (IRR = 0.63; 95% CI: 0.43, 0.90) and richest (IRR = 0.49; 95% CI: 0.28, 0.87) quartiles were associated with having a lower probability of smoking intensively. In Ethiopia, smoking intensity also significantly varied among regional states. Smokers in the Somali (IRR = 2.80; 95% CI: 1.29, 6.11), Harari (IRR = 3.46; 95% CI: 1.14, 10.51), and Dire Dawa (IRR = 3.09; 95% CI: 1.23, 7.80) regions smoked 2.8, 3.5, and 3.1 times greater number of cigarettes than those in the Tigray region. Smokers who watched television smoked 1.56 times more cigarettes than their counterparts (IRR = 1.18; 95% CI: 1.035, 1.346). On the other hand, men who take alcohol smoked 1.4 more number of cigarettes than their counterparts (IRR = 1.37; 95% CI: 1.025, 1.82) (see Table 3).

## 4. Discussion

This study explored smoking intensity and associated factors among male smokers in Ethiopia, using the national 2016 EDHS data. The finding showed that on average men smoked weighted nine cigarettes per day. This finding is a little higher than the report of a similar study in Ghana [15]. However, it is lower than daily smokers in the United States of America who consumed 14 cigarettes daily in 2016 [16]. This may be due to socioeconomic differences among nations.

Smokers living in rural areas smoked 57% fewer number of cigarettes than those living in urban areas. This finding is in line with a study done in Ethiopia [6] and Nepal [17] in which urban dwellers are more likely to smoke tobacco than rural residents. In contrast, a study in 30 sub-Saharan African countries found that rural residents are more likely to smoke than urban residents [18]. However, other studies revealed that place of residence is not a significant predictor of smoking [4, 8]. This could be explained by the fact the differences in the availability and accessibility of manufactured cigarettes between the urban and rural areas.

In our study, older daily smokers have a higher likelihood to smoke cigarettes intensively, with the older group (45–59 years) smoking 1.8 times more cigarettes than the younger counterparts (15–29 years). This study finding is similar with a study done in Ghana and Ethiopia [6], Harari [8], Ethiopia and Kenya [11], and Nepal [19], where younger men have a lower likelihood to smoke greater quantities of cigarettes. This may be due to the fact that older people had extended tobacco use and experience which increased the smoking intensity [4, 8]. So, policymakers and programmers need to select and implement interventions reaching elderly smokers.

In our study, currently married and formerly married men smoke 36% and 46% fewer cigarettes than those who never married, respectively. This study finding is different from EDHS-based studies done in Ethiopia [4, 6], Kenya [20], and Nepal [19], where formerly married and married individuals were more likely to smoke tobaccos compared to their not-married counterparts. On the other hand, though men who are affiliated with Protestant religion were found to be less likely to smoke, those who smoke have a higher likelihood to smoke a greater number of cigarettes per day than those who are affiliated with Islam religion. This finding is similar with an EHDS-based study in Ethiopia [6].

Smokers who completed primary and higher education smoke a higher number of cigarettes than those who were illiterate. This is similar with a study done in Ghana and different from studies done in Nepal [15, 17, 19]. Then again, compared to smokers in the poorest wealth quartile, those who were in the poorer quartile smoked 1.64 times more cigarettes. This may be due to the slight enhanced economic status of poorer men than the poorest men, which enables them to cover the cost of more cigarettes. Conversely, smokers in the richer and richest quartiles were associated with having a lower probability of smoking intensively. This finding is consistent with those reported in Ethiopia, Ghana, and Nepal [4, 6, 11, 15, 19]. Generally, improving the economic status of the community is one of a positive policy direction.

Smokers in the Somali, Harari, and Dire Dawa region smoked a greater number of cigarettes than those in the Tigray region of Ethiopia. This regional variation in smoking intensity was corroborated by other studies done on tobacco use in Ethiopia [4, 6]. The difference might be attributed to differences in demographics as well as religious and cultural practices. On the other hand, men who watched television smoked 1.56 times greater number of cigarettes than their counterparts. This finding is different from a study done in Nepal, where those who watched television at least once a week were less likely to consume tobacco products [19]. Men who take alcohol smoke 1.4 times greater number of cigarettes than their counterparts. This finding is similar with the study done in Kenya [20]. This study result revealed that tobacco control interventions need to be tailored to address other substance use behaviors.

### 4.1. Limitation of the Study

This study has its own limitations. First, it focused on men's daily cigarette consumption. Second, the data on the number of cigarettes smoked per day was self-reported which may have recall bias and social desirability bias, leading to an underreporting of the rates. Finally, the study used the number of manufactured cigarettes used per day to measure the smoking intensity.

## 5. Conclusion

Male smokers in Ethiopia smoked intensively with an average of nine manufactured cigarettes per day.

Being currently and formerly married, rural residence, and higher wealth status were associated with lower smoking intensity. Eastern Ethiopia regions, older age, affiliated with Protestant religion, poorer men, watching television, drinking alcohol, and primary and higher education were positively associated with smoking intensively. Since reducing intensity was a behavioral step towards cessation tobacco use, interventions should target the following: Eastern Ethiopia regions, older aged, affiliated with Protestant religion, poorer men, watched television, drunk alcohol, and primary and higher educational level.

## Figures and Tables

**Table 1:** Model comparison using Akaike Information Criterion and Bayesian Information Criterion.

S. no	Models	LL (model)	AIC	BIC
1.	Poisson regression	-1587.815	3245.63	3405.365
2.	Negative binomial regression	-1091.84	2255.68	2419.979
3.	Truncated Poisson regression	-1583.406	3236.813	3396.547
4.	Zero-inflated Poisson model regression	-1092.322	2258.644	2427.506
5.	Truncated negative binomial regression	-1068.039	2208.078	2372.377

**Table 2:** Average number of cigarettes smoked daily among male adult smokers across sociodemographic factors in Ethiopia, 2016 (*N* = 736).

Variables		Weighted frequency (%)	Mean cigarette smoked	Standard deviation of cigarettes smoked
Age group	15-29	85 (21.62)	10.3	1.80
	30-44	190 (48.71)	7.63	0.52
	45-59	116 (29.68)	9.77	1.17

Place of residence	Urban	91 (23.24)	13.10	1.90
	Rural	300 (76.76)	7.56	0.47

Marital status	Never married	56 (14.41)	12.46	2.53
	Currently married	322 (82.37)	8.26	0.54
	Formerly married	13 (3.22)	7.76	2.19

Religion	Muslim	270 (68.98)	8.14	0.53
	Orthodox	93 (23.81)	11.37	1.86
	Protestant	22 (5.63)	7.90	1.45
	Other	6 (1.58)	4.90	2.08

Educational level	No education	149 (38.23)	7.23	0.64
	Primary	164 (42.07)	7.78	0.63
	Secondary	45 (11.52)	8.36	1.19
	Higher	32 (8.18)	22.56	4.79

Occupational status	Not working	23 (5.76)	5.69	0.92
	Professional	73 (18.81)	11.91	1.99
	Agricultural	240 (61.45)	7.72	0.56
	Unskilled	18 (4.61)	14.29	4.99
	Other	37 (9.37)	9.38	1.38

Listen to radio	No	209 (53.55)	8.10	0.81
	Yes	182 (46.45)	9.75	0.83

Read newspaper	No	298 (76.18)	8.51	0.57
	Yes	93 (23.82)	9.94	1.62

Watch television	No	241 (61.55)	6.37	0.38
	Yes	150 (38.45)	12.81	1.33

Administrative region	Tigray	6 (1.54)	9.03	3.16
	Afar	4 (0.98)	14.31	12.61
	Amhara	18 (4.03)	15.55	5.59
	Oromia	202 (51.79)	9.38	0.93
	Somali	60 (15.31)	8.83	0.73
	Benishangul	10 (2.53)	6.94	1.98
	SNNPR	47 (12.14)	4.20	0.36
	Gambela	3 (0.87)	7.77	4.12
	Harari	4 (0.94)	13.31	4.50
	Addis Ababa	30 (7.74)	7.57	1.18
	Dire Dawa	8 (2.12)	12.70	4.12

Wealth index	Poorest	86 (22.14)	7.44	0.76
	Poorer	67 (17.10)	10.80	1.08
	Middle	87 (22.32)	7.13	1.09
	Richer	48 (12.31)	5.06	0.70
	Richest	102 (26.13)	12.01	1.70

Chewed khat	No	55 (14.78)	6.26	0.83
	Yes	317 (85.22)	9.14	0.68

Drunk alcohol	No	259 (69.40)	7.85	0.52
	Yes	114 (30.60)	10.68	1.52

**Table 3:** Truncated negative binomial Poisson regression model for the number of cigarettes smoked daily among male adult smokers in Ethiopia, 2016.

Variables (references)		IRR	Standard error	*p* value	95% CI
Residence (urban)	Rural	0.430	0.124	0.003	(0.244, 0.756)

Age category (15-29)	30-44	1.083	0.145	0.551	(0.834, 1.407)
	45-59	1.774	0.273	0.000	(1.313, 2.399)

Marital status (never married)	Currently married	0.644	0.113	0.012	(0.456, 0.909)
	Formerly married	0.538	0.160	0.037	(0.30, 0.964)

Religion (Muslim)	Orthodox	1.137	0.194	0.450	(0.814, 1.588)
	Protestant	1.808	0.442	0.015	(1.120, 2.918)
	Other	1.120	0.509	0.803	(0.460, 2.730)

Educational status (illiterate)	Primary	1.393	0.167	0.006	(1.101, 1.762)
	Secondary	1.438	0.270	0.053	(0.995, 2.076)
	Higher	2.959	0.689	0.000	(1.875, 4.669)

Occupation (not working)	Professional	1.246	0.310	0.376	(0.766, 2.029)
	Agricultural	1.494	0.357	0.093	(0.935, 2.386)
	Unskilled	1.080	0.332	0.803	(0.591, 1.971)
	Other	1.103	0.288	0.708	(0.661, 1.840)

Wealth index (poorest)	Poorer	1.644	0.271	0.003	(1.190, 2.272)
	Middle	0.841	0.134	0.277	(0.615, 1.149)
	Richer	0.626	0.117	0.012	(0.434, 0.904)
	Richest	0.491	0.142	0.014	(0.279, 0.866)

Administrative region	Afar	3.317	1.927	0.039	(1.062, 10.358)
	Amhara	1.179	0.526	0.712	(0.492, 2.826)
	Oromia	1.493	0.554	0.280	(0.722, 3.088)
	Somali	2.802	1.114	0.010	(1.286, 6.107)
	Benishangul	1.384	0.647	0.487	(0.553, 3.462)
	SNNPR	0.773	0.311	0.523	(0.351, 1.702)
	Gambela	0.851	0.513	0.789	(0.261, 2.772)
	Harari	3.464	1.962	0.028	(1.141, 10.514)
	Addis Ababa	0.653	0.262	0.289	(0.297, 1.435)
	Dire Dawa	3.094	1.460	0.017	(1.227, 7.804)

Hear radio (no)	Yes	1.242	0.141	0.056	(0.994, 1.551)
Watch television (no)	Yes	1.557	0.204	0.001	(1.205, 2.012)
Read newspapers (no)	Yes	0.822	0.112	0.149	(0.629, 1.073)
Alcohol use (no)	Yes	1.367	0.200	0.034	(1.025, 1.820)
Chewed khat	Yes	1.192	0.201	0.298	(0.856, 1.659)
Constant		4.313	2.244	0.005	(1.556, 11.957)
Alpha		0.5348	0.062		(0.424, 0.671)
Ln (alpha)		-0.628	0.117		(-0.857, -0.398)

∗Statistically significant at *p* value of <0.05.

## Data Availability

The data can be available upon request to the corresponding author.

## Abbreviations

AIC: Akaike Information Criterion
BIC: Bayesian Information Criterion
CDC: Centers for Disease Control and Prevention
CI: Confidence interval
CSA: Central Statistical Agency
DHS: Demographic and Heath Surveys
IRR: Incidence rate ratio
NCDs: Noncommunicable diseases
SNNPR: Southern Nations, Nationalities, and People's Region
WHO: World Health Organization.
